# Intratympanic steroid administration and predictors of recovery in sudden sensorineural hearing loss

**DOI:** 10.1371/journal.pone.0332809

**Published:** 2025-10-09

**Authors:** Devin J. Kennedy, Erin Williams, Lucienna Wolf, Jacquelyn Golden, Addison Lana, Matthew Wiefels, Madeline Pyle, Valerie Yunis, Michael Hoffer

**Affiliations:** 1 Department of Otolaryngology, University of Miami Miller School of Medicine, Miami, Florida, United States of America; 2 Department of Biomedical Engineering, University of Miami, Coral Gables, Florida, United States of America; 3 Department of Neurosurgical Surgery, University of Miami Miller School of Medicine, Miami, Florida, United States of America; University of Minnesota Twin Cities Department of Medicine, UNITED STATES OF AMERICA

## Abstract

**Objectives:**

Sudden sensorineural hearing loss patient outcomes remain highly variable despite established treatment modalities. This study investigates the effects of treatment modalities, individual-level risk factors, and clinical presentation on sudden sensorineural hearing loss recovery.

**Methods:**

Retrospective chart review of n = 231 patients who initiated intratympanic steroids between 01/01/2015-01/01/2025 following sudden sensorineural hearing loss. We collected individual-level risk factors and comprehensive audiometric data. Logistic and linear regression analyses were performed to identify relationships between patient, disease, and treatment-related variables to audiometric recovery (pure-tone average and word recognition score). Segmented linear regression analysis was used to assess the influence of intratympanic steroid treatment delay on patient recovery. Recovery outcomes were determined in accordance with American Academy of Otolaryngology-Head and Neck Surgery criteria.

**Results:**

Earlier intratympanic steroid initiation following sudden sensorineural hearing loss symptom onset significantly predicted increased pure-tone average recovery (p < 0.001) irrespective of intratympanic steroid status as primary treatment or as salvage therapy following oral steroids. Intratympanic steroid efficacy significantly diminished if initiated beyond 18 days following symptom onset (p < 0.001). Total number of intratympanic received and time between subsequent intratympanic injections did not impact recovery. Patient-level factors including demographics, socioeconomic status, medical and social risk factors, and medical comorbidities were largely found to have no significant relationship with recovery. Increased initial pure-tone average was associated with poorer overall pure-tone average recovery (p < 0.001). The presence of initial serviceable hearing predicted worse final recovery status (p = 0.01). Bilateral sudden sensorineural hearing loss showed significantly worse pure-tone average recovery compared to unilateral sudden sensorineural hearing loss (p = 0.04).

**Conclusion:**

Intratympanic steroids are an effective treatment option for sudden sensorineural hearing loss. Clinicians should be mindful of intratympanic steroid initiation timing and case-specific factors that may influence sudden sensorineural hearing loss recovery when treating patients with sudden sensorineural hearing loss.

## Introduction

Sudden sensorineural hearing loss (SSNHL) is an emergent otologic condition with approximately 66,000 new cases reported in the United States each year [1]. Clinically described as an acute loss of sensorineural hearing within a 72-hour period affecting one or both ears, the diagnosis of SSNHL is determined through a combination of patient history and audiometric testing.

Although standard diagnostic criteria for SSNHL constitutes a decrease of ≥30 decibels in hearing thresholds affecting three or more consecutive frequencies, many clinicians have adopted more flexible cutoffs to prioritize symptomatic complaints and overall clinical picture of their patients [2]. Current clinical practice guidelines for SSNHL include the administration of oral, systemic corticosteroids and/or salvage intratympanic (IT) steroid injections [2], though they have been shown to be comparatively efficacious when given adjunctively [3]. Despite these established treatment paradigms, SSNHL hearing outcomes remain highly variable. Conclusive assessment of steroid efficacy has been hindered by a limited understanding of SSNHL pathogenesis and study design heterogeneity [4].

To date, no prior studies have investigated treatment timing, patient-level risk factors, and both primary and salvage therapy in the context of outcomes following SSNHL. This retrospective cohort study addresses that gap by evaluating hearing outcomes with relation to established SSNHL treatments while also incorporating underexplored, patient-level factors – including demographics, social determinants of health, comorbidities, psychosocial risk factors, and SSNHL case-specific findings – that may influence treatment efficacy and recovery trajectories.

## Materials and methods

### Retrospective chart review

**Fig 1:**
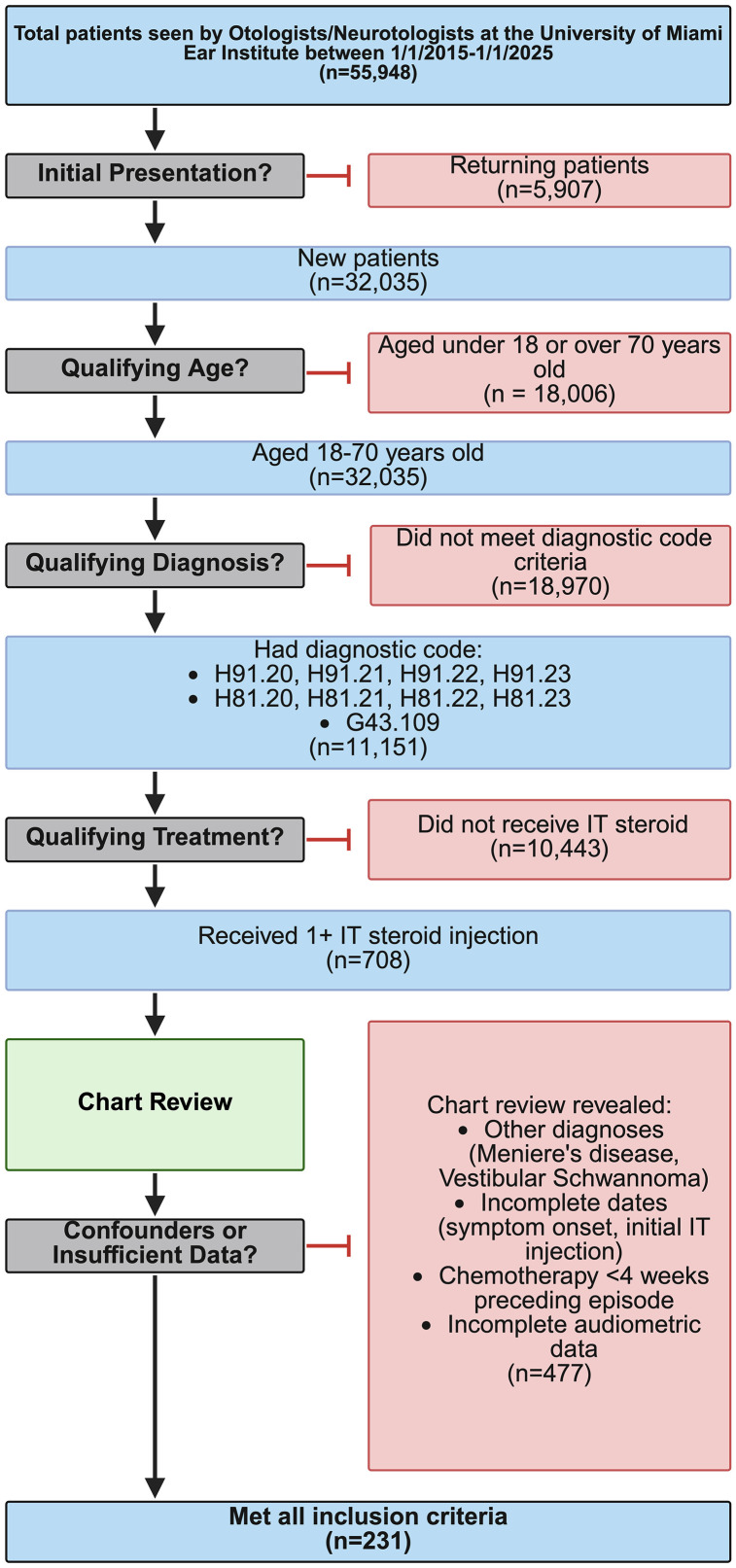
Consort diagram. SSNHL patients were identified according to pre-specified criteria. Patients were excluded from further chart review and analysis if they received additional diagnoses of vestibular schwannoma or Meniere’s disease, chemotherapy within 4 weeks of symptom onset, did not have record of symptom onset or initial IT date, or had incomplete audiometric data.

We reviewed the experience of seven otologists/neurotologists at an urban tertiary care academic medical center over a ten-year period of time from 01/01/2015–01/01/2025 and identified an initial n = 55,948 total patients. Only n = 708 patients met eligibility criteria for retrospective chart review. Eligible patients presented at the Ear Institute with SSNHL and received at least one intratympanic (IT) injection of steroid (CPT#: 69801) at the discretion of the treating physician. Inclusion criteria stipulated that patients be between 18−70 years of age and must have received their first IT injection between 01/01/2015-01/01/2025. Patients older than 70 years were excluded to minimize confounding from presbycusis. In this study, IT injections administered more than 6 months following initial IT injection were considered outside the initial IT treatment series and were excluded from analysis. Similarly, patients who received chemotherapy treatment within 4 weeks preceding symptom onset and those with a diagnosis of vestibular schwannoma or Meniere’s disease were also excluded. Lastly, individuals without complete initial and final pure-tone thresholds at all four specified frequencies or were missing symptom onset or initial IT dates were likewise excluded. Following chart review, n = 231 total patients met all inclusion criteria and no exclusion criteria that were ultimately included for analysis (Fig 1). This review was approved by the IRB at the University of Miami (IBIS#20230698).

Charts were reviewed for demographics (i.e., age, postal code, sex, race, and ethnicity), comorbidities, lifestyle-related risk factors, and medications. Postal codes were cross-referenced with National Census data [5] to collect median household income, health insurance coverage, and employment rate data for each patient. National Census data was used to stratify patients into socioeconomic categories based on predetermined criteria that can be found in Fig 1. Social risk factors included any history of tobacco smoking, smokeless tobacco, vaping, alcohol use, recent loud noise exposure, lifetime noise exposure, or recent aural trauma. Medical risk factors included changes in any of the following factors with reasonable proximity to SSNHL (i.e., medications, intravenous antibiotics, chemotherapy, illness/vaccinations. Comorbidities were documented both individually and across functional systems, including cardiovascular, pulmonary, neurological, autoimmune, endocrine, and psychiatric disease. Diagnoses outside of these discrete groupings were categorized as “other”.

**Fig 2:**
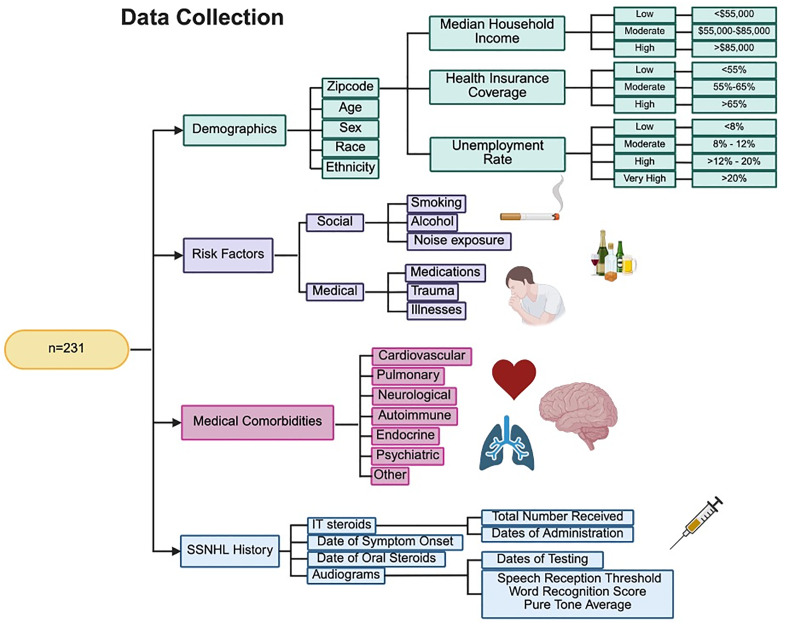
Data collection flow chart. Data collection methodology including demographic, risk factor, medical comorbidity, and SSNHL information.

Detailed information pertaining to each patient’s acute SSNHL episode and subsequent clinical course was collected, including symptom onset date, affected ear/sidedness, IT injection dates, and oral steroids. Audiometric data for each ear, including word recognition scores (WRS) and pure tones at 0.5, 1, 2, and 3 kHz, were collected at two timepoints, at the visit closest to or during initial IT administration and again following final IT injection. All audiometric measurements were obtained by a licensed clinical audiologist with calibrated, clinically operated equipment. Pure tone averages (PTA) were calculated at each timepoint. An overview of data collected from patient charts can be found in Fig 2.

### Outcomes

Hearing serviceability and recovery classifications were classified according to the American Academy of Otolaryngology-Head and Neck Surgery (AAO-HNS) guidelines [2]. Serviceable hearing was defined as a PTA of ≤50 dB and a WRS of ≥50%. Patient recovery classifications were based on audiometric outcomes, and included complete recovery (final PTA ≤ 10 dB and WRS ≤ 10% relative to unaffected side), and partial recovery (either final PTA improvement to ≤50 dB and WRS to ≥50% if the affected ear was initially unserviceable, or a ≥ 10 dB improvement in PTA or a ≥ 10% improvement in WRS if the ear was initially serviceable). Patients who did not meet criteria for either complete or partial recovery were classified as having no response. Due to the inability to assess improvement relative to an unaffected side, bilateral SSNHL recovery was based on averaged PTA and WRS data across both ears, with recovery defined as complete (final PTA ≤ 25 dB), partial (improvement of ≥10 dB in PTA or ≥10% in WRS), or no response.

### Statistical analysis

Segmented linear regression was used to assess whether the association between time to intratympanic (IT) steroid administration and hearing outcomes exhibited a threshold effect. Given clinical observations and prior literature suggesting a time-sensitive therapeutic window for steroid efficacy in SSNHL, we hypothesized that the relationship between treatment delay and hearing recovery would be non-linear across the entire range of delays. Therefore, we fitted piecewise linear models with a single breakpoint using the segmented package in R. The breakpoint represents the estimated value of days elapsed from symptom onset to first IT injection at which the slope of the association with hearing change significantly shifts. Breakpoint was initially estimated using clinical heuristics and then refined iteratively via bootstrapping within the model-fitting procedure.

To assess predictors of clinical outcomes following SSNHL, we conducted both logistic and linear regression analyses. Recovery was first modeled as a binary outcome using logistic regression, where patients were classified as either having recovered (i.e., partial or complete recovery) or not, as per the outcome definitions described above. Hearing improvement was defined as the change in PTA in the affected ear from initial presentation to final follow-up, with more negative values indicating greater improvement. This approach allowed for examination of the likelihood of recovery as a function of demographic, socioeconomic, and clinical variables. To complement this categorical outcome, we also modeled continuous hearing change by examining the change in PTA in the affected ear following treatment. All regression models included relevant predictors grouped into five thematic blocks: demographics, socioeconomic status, medical comorbidities, risk factors, and SSNHL progression and treatment. Predictors were selected based on prior literature and clinical relevance. All statistical tests were two-tailed with α = 0.05. All analyses were conducted using R (Version 2024.12.1 + 563).

## Results

### Cohort characteristics

A summary of patient demographics, social determinants of health, risk factors, medical comorbidities, and SSNHL clinical data are listed in Table 1.

**Table 1:** Summarized descriptive statistics of demographic, social determinants of health, risk factors, comorbidities, and detailed SSNHL-specific findings.

Demographics, Social Determinants of Health, Risk Factors, Comorbidities, SSNHL History			
**Demographics**	Sex	Male	111 (48.1%)
		Female	120 (51.9%)
	Race	Asian	7 (3.0%)
		Black or African American	11 (4.8%)
		Pacific Islander	1 (0.4%)
		White	198 (85.7%)
		Two or More Races	4 (1.7%)
		Unknown or Not Reported	10 (4.3%%)
	Ethnicity	Hispanic or Latino	114 (49.4%)
		Non-Hispanic or Latino	99 (42.9%)
		Unknown or Not Reported	18 (7.8%)
	Age in years (SD)	51.0 (±12.6)	
**Social Determinants of Health**	Median Household Income	Low	30 (13.3%)
		Moderate	106 (46.9%)
		High	90 (39.8%)
	No Health Insurance Coverage	Low	58 (25.7%)
		Moderate	70 (31.0%)
		High	64 (28.3%)
		Very High	34 (15.0%)
	Employment Rate	Low	46 (20.4%)
		Moderate	130 (57.5%)
		High	50 (22.1%)
**Risk Factors**	Smoking	Tobacco	60 (26.0%)
		Smokeless Tobacco	4 (1.7%)
		Vape	3 (1.3%)
	Noise Exposure	Chronic	29 (12.6%)
		Recent	22 (9.5%)
	Alcohol	127 (55.0%)	
	Recent Medication Change	24 (10.4%)	
	Recent Illness	46 (19.9%)	
	Recent IV Antibiotics	9 (3.9%)	
	Recent Vaccination	8 (3.5%)	
	Recent Aural Trauma	8 (3.5%)	
**Medical Comorbidities**	Cardiovascular	107 (46.3%)	
	Pulmonary	29 (12.6%)	
	Neurological	45 (19.5%)	
	Autoimmune	27 (11.7%)	
	Endocrine	51 (22.1%)	
	Psychiatric	31 (13.4%)	
	Other	107 (46.3%)	
**SSNHL History**	Affected Side(s)	Right	96 (41.6%)
		Left	120 (51.9%)
		Both	15 (6.5%)
	Unilateral PTA	Initial (Affected)	64.5 dB (±42.6)
		Initial (Unaffected)	13.3 dB (±22.7)
		Final (Affected)	52.6 dB (±42.8)
		Final (Unaffected)	12.2 dB (±20.7)
	Bilateral PTA	Initial	45.1 dB (±32.5)
		Final	50.2 dB (±41.0)
	Unilateral WRS	Initial (Affected)	55.0% (±42.3)
		Initial (Unaffected)	95.2% (±15.9)
		Final (Affected)	66.3% (±39.8)
		Final (Unaffected)	96.2% (±14.5)
	Bilateral WRS	Initial	80.1% (±24.9)
		Final	76.3% (±30.0)
	Serviceable Hearing	Initial	87 (40.3%)
		Final	116 (53.7%)
	Oral Steroid Administered	Yes	177 (76.6%)
		No	54 (23.4%)
	Oral Steroid Timing	Pre-IT	92 (39.8%)
		Concurrent	81 (35.1%)
		Post-IT	3 (1.3%)
		None/No Record	55 (23.8%)
	IT Injections	Total Number of IT Received	2.8 (±1.3)
		Number Received 1 IT	36 (15.6%)
		Number Received 2 IT	54 (23.4%)
		Number Received 3 IT	94 (40.7%)
		Number Received 4 + IT	47 (20.3%)
		Days to First IT	29.6 (±32.3)
		Days Between IT #1 and IT #2	7.3 (±4.6)
		Days Between IT #2 and IT #3	7.6 (±5.3)
		Days Between IT #3 and IT #4	11.9 (±10.9)
	Recovery	Complete	63 (27.3%)
		Partial	31 (13.4%)
		No Response	137 (59.3%)

Demographic information including patient age, sex, race and ethnicity. Social determinants of health including median household income, percentage without health insurance, and unemployment rates were collected by cross-referencing patient postal codes with publicly available National Census data and were categorized according to predetermined criteria shown in Fig 1. History of alcohol use and smoking were obtained from patient records. Recent (i.e., within 4-weeks preceding symptom onset) and chronic exposures were extracted from available medical and patient reports. Medical comorbidities were gathered and categorized by functional systems. SSNHL-specific information was collected pertaining to the timing of symptoms and IT/oral steroid treatment in addition to total number of IT injections received, hearing impairment sidedness, and audiometric data obtained from audiograms closest preceding treatment and closest following final IT injection.

### Treatment modalities

Linear regression was used to examine the association between post-treatment hearing improvement and the timing of systemic and intratympanic steroid administration, as well as their interaction. Predictor variables included days between symptom onset and first IT, systemic steroid administration timing relative to intratympanic treatment (categorized as before, concurrent with, after, or no systemic treatment), and the interaction between these two temporal variables to evaluate whether their combined effect influenced hearing outcomes. Average values for each treatment intervention stratified by recovery group are displayed in Table 2.

**Table 2:** Mean values of treatment interventions according to recovery group.

Recovery Group	No Response (N = 137)	Partial Recovery (N = 31)	Complete Recovery (N = 63)	Total (N = 231)
**Days From Symptom Onset To First Injection**	33.5 (±33.6)	22.6 (±22.0)	24.8 (±32.8)	29.7 (±32.3)
**Oral Steroid Time**				
Pre-ITJ	55 (52.9%)	13 (44.8%)	24 (48.0%)	92 (50.3%)
Concurrent	43 (41.3%)	15 (51.7%)	23 (46.0%)	81 (44.3%)
Post-ITJ	2 (1.9%)	1 (3.4%)	0 (0.0%)	3 (1.6%)
None	4 (3.8%)	0 (0.0%)	3 (6.0%)	7 (3.8%)
**Total Injections**	2.9 (±1.4)	3.1 (±1.4)	2.6 (±1.1)	2.8 (±1.3)

Average days to first IT injection and total injections categorized by recovery status. Recovery group-specific breakdown of oral steroids timing relative to first IT injection. Overall, non-responders (33.5 days) had much longer time from developing symptoms to IT initiation compared to the partial (22.6 days) and complete (24.8 days) recovery groups. Oral steroid timing and total number of injections received were largely similar between all three recovery groups.

A significant positive association was found between days elapsed following symptom onset to first IT injection and PTA change in the affected ear (β = 0.49, 95% CI [0.25,0.74], p < 0.001), indicating that delays in initiating IT steroids were associated with reduced hearing improvement. Additionally, compared to the reference group (i.e., concurrent IT administration), patients treated with oral steroids prior to IT (β = 12.35, SE = 5.64, t = 2.19, p = 0.03), patients treated with oral steroids following IT (β = 32.35, SE = 20.62, t = 1.57, p = 0.12), and patients who received no oral steroids (β = 15.11, SE = 18.30, t = 0.83, p = 0.41) had worse hearing outcomes as evidenced by a higher final PTA. Notably, this effect was only statistically significant for the pre-IT oral steroid group. No other main effects and interaction terms in this model were significant.

**Fig 3:**
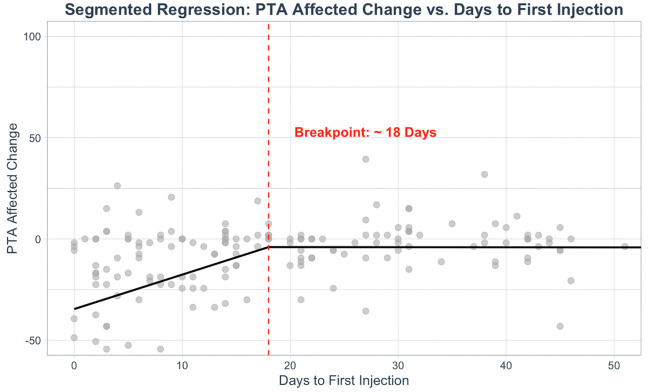
Treatment latency breakpoint independent of systemic steroid treatment. Segmented regression analysis revealed a significant breakpoint at 18 (SE: 2.92) days from SSNHL symptom onset to receipt of first IT steroid injection, where significantly worse PTA recovery was seen in patients initiating IT steroid treatment after this interval (p < 0.001).

Next, segmented linear regression was conducted to identify potential non-linear relationships between IT injection initiation and changes in hearing outcomes. We identified a breakpoint of 18 days from symptom onset to receipt of first IT injection, independent of systemic steroid treatment timing, where significantly worse audiometric recovery was observed among patients initiating IT steroid treatment after this interval (β = 18.0, SE = 3.0). Prior to this breakpoint, each additional day of delay in intratympanic treatment was associated with a significant worsening in hearing outcomes (β = 1.95, SE = 0.41, p < 0.001). However, the slope reversed after the breakpoint (Δβ = −1.93, SE = 0.42), suggesting that the benefit of IT initiation plateaued beyond this point (Fig 3). The segmented model explained a moderate proportion of variance in hearing outcomes (adjusted R² = 0.22), substantially improving fit compared to the unsegmented model (adjusted R² = 0.08). Additional analyses examining the number of days to first IT, the relative timing of systemic steroid administration (before, concurrent with, after, or no systemic treatment), and their interaction are provided in S1 and S2 Tables.

### Demographics

Logistic regression examined associations between demographic characteristics and the likelihood of recovery following IT treatment. No individual predictors showed statistically detectable associations with recovery (S3 Table). There were also no significant associations between socioeconomic indicators and recovery status. Employment rank demonstrated a trend toward association, (β = 0.62, SE = 0.36, z = 1.72, p = 0.09).

Similarly, in a simple linear regression (S2 Table) predicting PTA change from the same demographic variables, no individual covariate was significantly associated with treatment outcomes. Increasing age at diagnosis had a small, non-significant effect, and male sex approached but did not reach statistical significance. Patients residing in areas with a moderate unemployment rate were significantly associated with greater improvement in PTA following treatment (β = −9.78, SE = 4.55, t = −2.15, p = 0.03).

### Comorbidities and risk factors

No individual medical comorbidity significantly predicted recovery, including cardiovascular disease or pulmonary disease (S3 Table). We also examined the association between behavioral and environmental risk factors in relation to recovery status, where only recent loud noise exposure trended towards significance (β = 1.00, SE = 0.51, z = 1.95, p = 0.052).

A linear regression associating recovery to medical comorbidities likewise revealed no statistically detectable patterns. Curiously, the only PTA-influencing behavioral or environmental risk factor was vaping, which had a positive association with improved hearing outcomes (β = −29.04, SE = 15.99, t = −1.82, p = 0.07).

### SSNHL progression and treatment

Multivariable logistic regression was used to examine clinical severity and treatment dynamics associated with recovery status. Higher baseline PTAs in the affected ear were negatively associated with partial or complete recovery (β = −0.06, SE = 0.01, z = −5.13, p < 0.001), as was longer time elapsed before the first injection (β = −0.02, SE = 0.01, z = −3.39, p < 0.001). Serviceable hearing at baseline also predicted lower odds of recovery (β = −1.86, SE = 0.66, z = −2.80, p = 0.005). Linear models were thusly constructed to assess the predictive value of SSNHL-related variables on changes in PTA following IT treatment. Longer delays before treatment were again associated with smaller changes in PTA (β = 0.24, SE = 0.05, t = 4.44, p < 0.001), as were higher baseline WRS scores (β = 0.17, SE = 0.08, t = 2.07, p = 0.04). All other variables were not statistically significant predictors in either model.

We also found that the independent effect of time between successive intratympanic injections, when holding all other variables constant, demonstrated no significant effect on recovery status (S3 Table). Similarly, none of the intervals significantly predicted improvement in hearing thresholds, with the 1^st^ to 2^nd^ interval, the 2^nd^ to 3^rd^, or the 3^rd^ to 4^th^ interval (S2 Table).

Finally, we performed a Wilcoxon rank sum test with continuity correction to compare the difference between PTA improvement in unilateral SSNHL versus bilateral SSNHL. Here, we observed a statistically significant negative effect among bilateral cases (W = 921.00, p = 0.036; r = −0.34, 95% CI [−0.58,-0.04]), indicating that patients with bilateral treatment tended to experience smaller improvements in PTA compared to unilateral cases.

## Discussion

This retrospective study included a mix of patients treated with IT steroids, either as primary or salvage therapy. On average, patients had PTA improvement of 12 dB and WRS improvement of 11% following treatment. Despite these improvements, only 41% of patients exhibited partial or complete recovery according to AAO-HNS guidelines [2], with nearly 60% of patients exhibiting no response to treatment. While these percentages align with previous reports, such as those from Parnes et al. who reported improvements in approximately one-third of patients following IT steroids [6], the reason for variability in SSNHL patient outcomes following IT treatment remains unclear.

We assessed several key variables related to IT management, including interval to initial intervention. Work by Slattery et. al found that IT steroid salvage therapy may be effective up to 3 months following symptom onset [7], though to date there has been limited evidence in support of an absolute time cutoff [2]. Notably, this investigation supports an IT treatment “breakpoint” of approximately 18 days, after which PTA recovery plateaued. These findings support current AAO-HNS guidelines for ideal IT therapy, which stipulate treatment within 14 days of SSNHL symptom onset. More broadly, this finding underscores the critical, time-sensitive nature of early referral and intervention, particularly among non-otolaryngologists [2,8,9].

The impact of other treatment variables, including total injections, time between serial injections, and timing of treatment relative to oral steroids remain controversial. Clinical practice is often institution or provider specific, though in general treatment consists of weekly IT injections for a total of 3–5 injections. Interestingly, our work supports Haynes et. al, where total number of IT injections received did not significantly change hearing outcomes [10]. Further, while the average time between subsequent IT injections approximated 7 days, the standard duration commonly used in practice, we found that time elapsed between injections did not result in prognostic differences.

Our patients recovered at a rate consistent with prior work [11,12], with 40% exhibiting partial or complete recovery when treated with IT steroids following oral steroid treatment. This number rose to 47% in patients treated with combination oral and IT steroids as their primary treatment. Ultimately, however, overall hearing outcomes were not significantly different amongst patients initially treated with oral steroids who then received IT salvage therapy, patients who received concurrent treatment with both oral and IT steroids, or in patients treated with IT steroids alone. The authors note that a key limitation of the overall interpretability of these results is that individuals who recovered following oral steroid treatment alone were not captured in this analysis. As such, direct comparison of IT efficacy relative to systemic therapy cannot be fully established from this cohort. In addition, the retrospective design raises the possibility of referral bias, as late-presenting patients may differ systematically from those who present earlier, which could influence the observed treatment outcomes. Furthermore, the possible influence of unmeasured confounders, such as adherence to oral steroid regimens or prior medical care before presentation, cannot be excluded. Nonetheless, our findings broadly support the use of IT steroids in the treatment of SSNHL, with a caveat that timing relative to oral steroid administration may be less critical than their timely administration itself.

Finally, contrary to previous work [13], we found that worse baseline PTA values predicted poorer recovery. The average time from symptom onset to IT treatment in our cohort was two weeks longer than those reported by Liebau et. al., which the authors suspect contributed to the discrepancy in recovery outcomes between cohorts. The population described herein likely benefited less from spontaneous recovery, which occurs within 14 days from symptom onset, and as a consequence are associated with worse hearing recovery [14–17].

While the above findings offer insight into clinical management, a secondary goal of this study was to identify patient-specific factors that may inform individualized SSNHL treatment. We examined hearing outcomes across a range of demographic, socioeconomic, clinical, and health-related domains, including age, sex, and medical comorbidities. Age was not found to significantly influence outcomes, nor were any other demographic variables, indicating that IT treatment may offer comparable benefits across diverse patient populations. Further, while social determinants of health investigated in this study also largely had no effect on audiometric outcomes, we did find that patients who lived in areas with a moderate (8–12%) unemployment rate predicted better recovery of PTA (p = 0.03). While the underlying reason for this finding is unclear, it is conceivable that employment status could influence patient well-being and lifestyle choices that may affect treatment efficacy. However, this association may represent an incidental finding. No other comorbidities or other social risk factors were found to be significant predictors of SSNHL recovery.

## Conclusion

This study evaluated the effectiveness of IT steroid therapy following SSNHL in a diverse patient population. Our findings demonstrated modest improvements in PTA and WRS and are generally supportive of IT steroids as a viable treatment option even in the absence of oral steroids. Earlier initiation of IT therapy was associated with better outcomes and underscores the critical importance of early intervention. Other treatment factors (i.e., total IT injections and their chronology relative to oral steroid use) did not significantly impact recovery, suggesting that timely delivery may be more critical than institution- or clinician-specific nuances in treatment paradigms.

## Supporting information

S1 Table Initial hearing serviceability, PTA, and WRS according to final recovery status. (DOCX)

S2 Table Linear regression analysis of factors predicting PTA change. (DOCX)

S3 Table Generalized linear model analysis of factors predicting recovery. (DOCX)

S1 File Data. (XLS)
