# Supplementary material for: Intratympanic steroid administration and predictors of recovery in sudden sensorineural hearing loss
Source: PLoS One. 2025 Oct 9;20(10):e0332809. doi: 10.1371/journal.pone.0332809 (PMC12510503; doi:10.1371/journal.pone.0332809)
Supplement: S1 Table — (DOCX) [file pone.0332809.s001.docx]

| **Recovery Group** | **No Response (N=137)** | **Partial Recovery (N=31)** | **Complete Recovery (N=63)** | **Total (N=231)** |
| --- | --- | --- | --- | --- |
| **Initial Serviceable Hearing** | 40 (30.5%) | 8 (28.6) | 39 (68.4%) | 87 (40.3%) |
| **Initial PTA** | 78.8 dB (±41.2) | 62.1 dB (±30.4) | 32.8 dB (±32.7) | 64.5 dB (±42.6) |
| **Initial WRS** | 44.7% (±43.0) | 55.6% (±33.3) | 78.4% (±35.2) | 55.0% (±42.3) |

**Table S1.** **Initial Hearing Serviceability, PTA, and WRS According to Final Recovery Status**. Proportion of patients presenting with serviceable hearing and initial PTA and WRS according to final recovery status.
