# Supplementary material for: Intratympanic steroid administration and predictors of recovery in sudden sensorineural hearing loss
Source: PLoS One. 2025 Oct 9;20(10):e0332809. doi: 10.1371/journal.pone.0332809 (PMC12510503; doi:10.1371/journal.pone.0332809)
Supplement: S2 Table — (DOCX) [file pone.0332809.s002.docx]

| **LM: PTA Change** | | | | | |
| --- | --- | --- | --- | --- | --- |
| **Category** | **Variable** | **Estimate** | **Standard Error** | **t-value** | **p-value** |
| **Demographics** | *Intercept* | -40.16 | 14.38 | -2.79 | 0.006 |
|  | Age | 0.15 | 0.17 | 0.87 | 0.38 |
|  | Sex (male) | -7.94 | 4.33 | -1.83 | 0.07 |
|  | Black or African American | 20.02 | 14.57 | 1.37 | 0.17 |
|  | Two or More Races | 30.32 | 22.01 | 1.38 | 0.17 |
|  | Unknown Race | 20.66 | 15.21 | 1.36 | 0.18 |
|  | White Race | 21.50 | 11.16 | 1.93 | 0.06 |
|  | Non-Hispanic or Latino | 4.38 | 4.58 | 0.96 | 0.34 |
|  | Unknown Ethnicity | 5.03 | 9.51 | 0.53 | 0.60 |
|  | | | | | |
| **Social Determinants of Health** | *Intercept* | -3.78 | 6.22 | -0.61 | 0.54 |
|  | Low Income | -2.12 | 7.77 | -0.27 | 0.79 |
|  | Moderate Income | 1.90 | 4.59 | 0.39 | 0.70 |
|  | Low Employment | -2.45 | 6.03 | -0.41 | 0.69 |
|  | Moderate Employment | -9.78 | 4.55 | -2.15 | **0.03*** |
|  | Low Uninsured | -2.74 | 6.41 | -0.43 | 0.67 |
|  | Moderate Uninsured | -6.94 | 5.14 | -1.35 | 0.18 |
|  | Very High Uninsured | -7.14 | 6.16 | -1.16 | 0.25 |
|  | | | | | |
| **Medical Comorbidities** | *Intercept* | -15.66 | 2.95 | -5.31 | <0.001*** |
|  | Cardiovascular | -1.67 | 3.74 | -0.45 | 0.66 |
|  | Pulmonary | -4.13 | 5.59 | -0.74 | 0.46 |
|  | Neurological | -1.51 | 4.85 | -0.31 | 0.76 |
|  | Autoimmune | -4.94 | 5.91 | -0.84 | 0.40 |
|  | Endocrine | -1.27 | 4.72 | -0.27 | 0.79 |
|  | Psychiatric | 1.06 | 5.56 | 0.19 | 0.85 |
|  | Other | 5.57 | 3.82 | 1.46 | 0.15 |
|  | | | | | |
| **Risk Factors** | *Intercept* | -12.25 | 3.07 | -4.00 | <0.001*** |
|  | Tobacco | 2.17 | 4.32 | 0.50 | 0.62 |
|  | Smokeless Tobacco | -20.42 | 13.99 | -1.46 | 0.15 |
|  | Vaping | -29.04 | 15.99 | -1.82 | 0.07 |
|  | Alcohol | -2.06 | 3.73 | -0.55 | 0.58 |
|  | Chronic Noise | 4.76 | 5.79 | 0.82 | 0.41 |
|  | Recent Noise | -2.58 | 6.42 | -0.40 | 0.69 |
|  | Recent Medication Change | 7.38 | 6.26 | 1.18 | 0.24 |
|  | Recent IV Antibiotics | -3.30 | 11.45 | -0.29 | 0.77 |
|  | Recent Chemotherapy | -0.23 | 17.15 | -0.01 | 0.99 |
|  | Recent Vaccination | -17.61 | 10.97 | -1.61 | 0.11 |
|  | Recent Aural Trauma | -0.78 | 9.45 | -0.08 | 0.93 |
|  | | | | | |
| **SSNHL-specific** | *Intercept* | -32.51 | 10.53 | -3.09 | 0.002** |
|  | Right Sided | -3.57 | 3.27 | -1.09 | 0.28 |
|  | Initial WRS | 0.17 | 0.08 | 2.07 | **0.04*** |
|  | Initial PTA | 0.01 | 0.08 | 0.16 | 0.87 |
|  | Total IT Received | 0.47 | 1.28 | 0.37 | 0.71 |
|  | Days to First IT | 0.24 | 0.05 | 4.44 | **<0.001***** |
|  | Initial Serviceable Hearing | 6.64 | 5.97 | 1.11 | 0.27 |
|  |  | | | | |
|  | *Intercept* | -17.96 | 3.12 | -5.76 | **<0.001***** |
|  | No Oral Steroids | 9.66 | 10.73 | 0.90 | 0.37 |
|  | Pre-IT Oral Steroids | 7.26 | 4.26 | 1.24 | 0.09 |
|  | Post-IT Oral Steroids | 19.84 | 15.99 | 1.71 | 0.22 |
|  |  | | | | |
|  | *Intercept* | -7.98 | 10.17 | -0.78 | 0.44 |
|  | Days between IT #1-2 | -1.77 | 1.59 | -1.11 | 0.28 |
|  | Days between IT #2-3 | -0.12 | 0.88 | -0.14 | 0.89 |
|  | Days between IT #3-4 | 0.05 | 0.38 | 0.12 | 0.91 |

**Table S2.** **Linear Regression Analysis of Factors Predicting PTA Change**. Linear regression analysis of patient factors including demographics, social determinants of health, medical comorbidities, risk factors, and SSNHL-specific categories and their relationship with PTA change.
