# Supplementary material for: Intratympanic steroid administration and predictors of recovery in sudden sensorineural hearing loss
Source: PLoS One. 2025 Oct 9;20(10):e0332809. doi: 10.1371/journal.pone.0332809 (PMC12510503; doi:10.1371/journal.pone.0332809)
Supplement: S3 Table — (DOCX) [file pone.0332809.s003.docx]

| **GLM: Recovery vs. No Recovery** | | | | | |
| --- | --- | --- | --- | --- | --- |
| **Category** | **Variable** | **Estimate** | **Standard Error** | **z-value** | **p-value** |
| **Demographics** | *Intercept* | -0.82 | 1.12 | -0.74 | 0.46 |
|  | Age | -0.002 | 0.01 | -0.19 | 0.85 |
|  | Sex (male) | 0.25 | 0.32 | 0.76 | 0.45 |
|  | Black or African American | 1.14 | 1.14 | 1.00 | 0.32 |
|  | Two or More Races | -14.42 | 1.02 | -0.01 | 0.99 |
|  | Unknown Race | 0.42 | 1.21 | 0.35 | 0.73 |
|  | White Race | 4.56 | 0.90 | 0.51 | 0.61 |
|  | Non-Hispanic or Latino | 0.25 | 0.34 | 0.73 | 0.46 |
|  | Unknown Ethnicity | -0.44 | 0.79 | -0.56 | 0.58 |
|  | | | | | |
| **Social Determinants of Health** | *Intercept* | -1.11 | 0.49 | -2.24 | 0.02 |
|  | Low Income | 0.52 | 0.59 | 0.88 | 0.38 |
|  | Moderate Income | -0.15 | 0.37 | -0.39 | 0.70 |
|  | Low Employment | 0.31 | 0.46 | 0.67 | 0.50 |
|  | Moderate Employment | 0.62 | 0.36 | 1.72 | 0.09 |
|  | Low Uninsured | 0.20 | 0.50 | 0.40 | 0.69 |
|  | Moderate Uninsured | 0.44 | 0.40 | 1.10 | 0.27 |
|  | Very High | 0.59 | 0.46 | 1.27 | 0.21 |
|  | | | | | |
| **Medical Comorbidities** | *Intercept* | -0.33 | 0.23 | -1.46 | 0.14 |
|  | Cardiovascular | 0.21 | 0.29 | 0.72 | 0.47 |
|  | Pulmonary | 0.21 | 0.44 | 0.49 | 0.63 |
|  | Neurological | 0.07 | 0.36 | 0.20 | 0.84 |
|  | Autoimmune | 0.49 | 0.44 | 1.10 | 0.27 |
|  | Endocrine | -0.15 | 0.36 | -0.41 | 0.68 |
|  | Psychiatric | 0.48 | 0.40 | 1.19 | 0.23 |
|  | Other | -0.19 | 0.29 | -0.64 | 0.52 |
|  | | | | | |
| **Risk Factors** | *Intercept* | -0.45 | 0.23 | -1.96 | 0.05 |
|  | Tobacco | -0.07 | 0.33 | -0.21 | 0.84 |
|  | Smokeless Tobacco | 0.86 | 1.11 | 0.78 | 0.44 |
|  | Vaping | -0.46 | 1.35 | -0.34 | 0.74 |
|  | Alcohol | 0.05 | 0.29 | 0.19 | 0.85 |
|  | Chronic Noise | -0.38 | 0.47 | -0.81 | 0.42 |
|  | Recent Noise | 1.00 | 0.51 | 1.95 | 0.05 |
|  | Recent Medication Change | -0.37 | 0.49 | -0.75 | 0.46 |
|  | Recent IV Antibiotics | 0.10 | 0.78 | 0.13 | 0.90 |
|  | Recent Chemotherapy | 1.34 | 1.38 | 0.97 | 0.33 |
|  | Recent Vaccination | 0.09 | 0.78 | 0.12 | 0.90 |
|  | Recent Aural Trauma | 0.39 | 0.73 | 0.53 | 0.60 |
|  | | | | | |
| **SSNHL-specific** | *Intercept* | 4.83 | 1.21 | 3.99 | <0.001*** |
|  | Right Sided | 0.10 | 0.35 | 0.29 | 0.77 |
|  | Initial WRS | -0.01 | 0.01 | -1.00 | 0.32 |
|  | Initial PTA | -0.06 | 0.01 | -5.13 | **<0.001***** |
|  | Total IT Received | 0.01 | 0.13 | 0.10 | 0.92 |
|  | Days to First IT | -0.02 | 0.01 | -3.39 | **<0.001***** |
|  | Initial Serviceable Hearing | -1.86 | 0.66 | -2.8 | **0.005**** |
|  |  | | | | |
|  | *Intercept* | -0.12 | 0.22 | -0.56 | 0.58 |
|  | No Oral Steroids | -0.16 | 0.80 | -0.21 | 0.84 |
|  | Pre-IT Oral Steroids | -0.27 | 0.31 | -0.46 | 0.38 |
|  | Post-IT Oral Steroids | -0.57 | 1.24 | -0.89 | 0.65 |
|  |  | | | | |
|  | *Intercept* | -0.76 | 0.86 | -0.88 | 0.38 |
|  | Days between IT #1-2 | -0.06 | 0.13 | -0.49 | 0.63 |
|  | Days between IT #2-3 | 0.06 | 0.06 | 0.97 | 0.33 |
|  | Days between IT #3-4 | 0.01 | 0.03 | 0.41 | 0.68 |

**Table S3. Generalized Linear Model Analysis of Factors Predicting Recovery**. Generalized linear model analysis of patient factors including demographics, social determinants of health, medical comorbidities, risk factors, and SSNHL-specific categories and their relationship with final recovery status.
